# Nandrolone decanoate interferes with testosterone biosynthesis altering blood–testis barrier components

**DOI:** 10.1111/jcmm.13092

**Published:** 2017-02-28

**Authors:** Rosario Barone, Alessandro Pitruzzella, Antonella Marino Gammazza, Francesca Rappa, Monica Salerno, Fulvio Barone, Claudia Sangiorgi, Daniela D'Amico, Nicola Locorotondo, Francesca Di Gaudio, Luigi Cipolloni, Valentina Di Felice, Stefania Schiavone, Venerando Rapisarda, Gabriele Sani, Amos Tambo, Francesco Cappello, Emanuela Turillazzi, Cristoforo Pomara

**Affiliations:** ^1^ Department of Experimental Biomedicine and Clinical Neurosciences (BioNeC) University of Palermo Palermo Italy; ^2^ Euro‐Mediterranean Institute of Science and Technology (IEMEST) Palermo Italy; ^3^ Department of Neuroscience Mental Health and Sense Organs (Nesmos) Sapienza University of Rome Rome Italy; ^4^ Department of Clinical and Experimental Medicine Forensic Pathology University of Foggia Foggia Italy; ^5^ Department of Radiology Scientific Institute Hospital “Casa Sollievo della Sofferenza” San Giovanni Rotondo (FG) Italy; ^6^ Locorotondo Labs srl Palermo Italy; ^7^ Department of Pathobiology and Medical Biotechnology University of Palermo Palermo Italy; ^8^ Department of Forensic Pathology Sapienza University of Rome Rome Italy; ^9^ Department of Clinical and Experimental Medicine University of Foggia Foggia Itlay; ^10^ Occupational Medicine Department of Clinical and Experimental Medicine University of Catania Catania Italy; ^11^ Department of Anatomy University of Malta Msida Malta

**Keywords:** nandrolone decanoate, testosterone, blood–testis barrier, TJP1, MMP‐2, MMP‐9, MUC1

## Abstract

The aim of this study was to investigate whether nandrolone decanoate (ND) use affects testosterone production and testicular morphology in a model of trained and sedentary mice. A group of mice underwent endurance training while another set led a sedentary lifestyle and were freely mobile within cages. All experimental groups were treated with either ND or peanut oil at different doses for 6 weeks. Testosterone serum levels were measured *via* liquid chromatography–mass spectrometry. Western blot analysis and quantitative real‐time PCR were utilized to determine gene and protein expression levels of the primary enzymes implicated in testosterone biosynthesis and gene expression levels of the blood–testis barrier (BTB) components. Immunohistochemistry and immunofluorescence were conducted for testicular morphological evaluation. The study demonstrated that moderate to high doses of ND induced a diminished serum testosterone level and altered the expression level of the key steroidogenic enzymes involved in testosterone biosynthesis. At the morphological level, ND induced degradation of the BTB by targeting the tight junction protein‐1 (TJP1). ND stimulation deregulated metalloproteinase‐9, metalloproteinase‐2 (MMP‐2) and the tissue inhibitor of MMP‐2. Moreover, ND administration resulted in a mislocalization of mucin‐1. In conclusion, ND abuse induces a decline in testosterone production that is unable to regulate the internalization and redistribution of TJP1 and may induce the deregulation of other BTB constituents *via* the inhibition of MMP‐2. ND may well be considered as both a potential inducer of male infertility and a potential risk factor to a low endogenous bioavailable testosterone.

## Introduction

Nandrolone decanoate is a synthetic testosterone analogue considered one of the most commonly abused anabolic androgenic steroids (AAS) by adolescents and athletes. ND is alleged to promote an increase in muscle mass and improves both physical appearance and sporting performance 1. Nowadays, ND abuse is often associated with serious adverse effects, interfering with the musculoskeletal system, the endocrine system and the reproductive system 2. Moreover, ND may suppress the hypothalamic–pituitary–gonadal axis resulting in a decreased production of endogenous testosterone 3.

Testosterone is usually produced by testicular Leydig cells and its production is regulated by a neuroendocrine feedback mechanism which regulates the pulsatile release of luteinizing hormone (LH). The result is therefore activation or repression of the steroidogenic signalling cascade as well as gene transcription of key enzymes 4, 5. The steroidogenic enzymes involved in testosterone biosynthesis include steroidogenic acute regulatory protein (StAR), cholesterol side‐chain cleavage enzyme (CYP11A1), 3ß‐hydroxysteroid dehydrogenase (HSD3B1), 17α‐hydroxylase/17,20‐lyase (CYP17A1) and 17ß‐hydroxysteroid dehydrogenase 6. The role of these steroidogenic enzymes is further described in Table 1.

**Table 1:** The role of some steroidogenic enzymes

Steroidogenic enzymes	Abbreviation	Role
Steroidogenic acute regulatory	StAR	Transfers cholesterol to the inner membrane of mitochondria
Cholesterol side‐chain cleavage enzyme	CYP11A1	Converts cholesterol into pregnenolone within the mitochondria
3ß‐hydroxysteroid dehydrogenase	HSD3B1	Converts pregnenolone into progesterone
17α‐hydroxylase/17,20‐lyase	CYP17A1	Converts progesterone into androstenedione

Although ND is frequently abused in sports, there are limited animal studies which compare the relationship between physical activity/exercise with and without ND use. Shokri *et al*. 7 demonstrated that exercise associated with supraphysiological doses of ND in rats increased apoptosis in spermatogenic cells. In testes, germ cell development is supported by Sertoli cells that reside within the basal epithelial lining within the seminiferous epithelium 8. These cells create a specialized microenvironment through the formation of the BTB, preventing free passage of solutes, ions and water that might affect the development of germ cells 9. The BTB is formed by tight junctions (TJs), basal ectoplasmic specializations (ES) and desmosome–gap junctions (D‐GJs) that compartmentalize the seminiferous tubule into the basal and adluminal compartments 9.

The aim of this study was to investigate the effects of ND administration on testosterone biosynthesis in a mouse exercise model. Moreover, testis morphological alterations associated with the dysregulation of factors that confer BTB integrity will also be determined.

## Materials and methods

### Animals and animal care

This experiment was carried out on forty‐eight 3‐month‐old healthy male CD1 mice (46.1 ± 3.2 g body weight). The mice were housed in cages and maintained in an animal room with controlled lighting (12‐hrs light–dark cycle) and temperature (21 ± 1°C). Animals were allowed free access to standard food and water. After 1 week of acclimatization, the animals were assigned to one of the eight experimental groups described in Table 2.

**Table 2:** Experimental groups

Groups	Abbreviation	Nandrolone Decanoate/kg per week
Normal control mice	SED	—
Sedentary low dose of nandrolone secanoate	SED‐ND‐L	3.75 mg
Sedentary middle dose of nandrolone decanoate	SED‐ND‐M	10 mg
Sedentary high dose of nandrolone decanoate	SED‐ND‐H	20 mg
Trained control mice	TR	—
Trained low dose of nandrolone decanoate	TR‐ND‐L	3.75 mg
Trained middle dose of nandrolone decanoate	TR‐ND‐M	10 mg
Trained high dose of ND nandrolone decanoate	TR‐ND‐H	20 mg

Experiments on animals were performed at the Department of Human Anatomy, Faculty of Medicine and Surgery, University of Malta, Msida, Malta. The care and treatment of all animals were carried out in accordance with the EU Council Directive 86/609/EEC, the Animals Scientific Procedures Act 1986. All experimental protocols were approved by the Faculty of Medicine and Surgery Animal Care and Use Committee, University of Malta.

### Training protocol and nandrolone administration

A motorized treadmill (Exer 3/6, Columbus, OH, USA) was used to train the mice. The TR mice ran 5 days/week at a progressively increasing duration and intensity of training. During the first week, the mice ran for 15 min. at a speed of 10 m/min.; during the second and third weeks, the mice ran for 30 and 60 min., respectively, at the same speed; during the fourth and fifth weeks, the mice ran for 60 min. at a speed of 12 m/min. Finally, during the last week, the mice ran for 90 min. at a speed of 14 m/min. All the mice were weighed weekly.

Mice of all experimental groups were treated with intramuscular injections (IM) of ND (Sigma‐Aldrich, St. Louis, MO, USA) or peanut oil twice a week in the hindlimb for 6 weeks (Table 2). ND was dissolved in peanut oil with 10% of benzoic alcohol, and the dose of ND was selected according to the literature 10, 11, 12. Mice of the SED and TR groups (controls) were administered IM peanut oil and 10% benzoic alcohol. Forty‐eight hours after the last training session, mice were killed *via* cervical dislocation. The blood was collected in tubes and centrifuged and serum was stored at −80°C. Testes were dissected and preserved in liquid nitrogen or embedded in paraffin for morphological and molecular evaluation.

### Measurement of testosterone level with liquid chromatography–mass spectrometry

Testosterone levels were assessed by ‘Locorotondo Labs srl, Palermo’. Testosterone in serum was quantified using a validated method for the analysis in serum/plasma of testosterone by liquid chromatography–mass spectrometry (LC‐MS/MS). The method was performed as described previously 13. Total testosterone analysis in serum was performed in all experimental groups (n = 6 per group).

### Western blotting analysis

Testis homogenization was performed as described previously 14, 15. The membrane was incubated in a blocking solution containing 5% milk in Tris‐buffered saline (20 mM Tris, 137 mM NaCl, pH 7.6) containing 0.05% Tween‐20 (T‐TBS) for 1 hr. Next, the membrane was further incubated in a primary antibody overnight at 4°C (see Table 3). All the primary antibodies were diluted in T‐TBS containing 5% BSA and incubated overnight at 4°C. The following day, the membrane was washed with T‐TBS and incubated with an HRP‐conjugated secondary antibody (anti‐rabbit NA934V, or antimouse NA931; Amersham Biosciences, GE Healthcare Life Science, Pittsburgh, USA) diluted in T‐TBS containing 5% milk for 1 hr. The detection of the immunopositive bands was performed using ECL Western blotting detection reagent (Amersham Biosciences) according to the manufacturer's instructions.

**Table 3:** Primary Antibody used for WB, IHC and IF

Method	Antigen	Type and source	Clone	Supplier	Dilution
WB	CYP17A1	Rabbit polyclonal	M‐80	Santa Cruz Biotechnology, Inc. (Dallas, TX, USA)	1:1000
WB	HSD3B1	Goat polyclonal	P‐18	Santa Cruz Biotechnology	1:1000
WB	StAR	Rabbit polyclonal	FL‐285	Santa Cruz Biotechnology	1:1000
WB	CYP11A1	Rabbit polyclonal	H‐165	Santa Cruz Biotechnology	1:1000
WB	β‐Actin	Mouse monoclonal	C‐2	Santa Cruz Biotechnology	1:1000
IF	Tight Junction Protein‐1 (TJP1)	Rabbit polyclonal	Not specified	Sigma‐Aldrich	1:50
IF	MMP‐2	Rabbit polyclonal	H‐76	Santa Cruz Biotechnology	1:50
IF	TIMP‐2	Mouse monoclonal	Not specified	Millipore (Temecula, CA, USA)	1:50
IF	MMP‐9	Rabbit polyclonal	H‐129	Santa Cruz Biotechnology	1:50
IHC	MUC1	Rabbit polyclonal	AB2	Sigma‐Aldrich	1:200

Abbreviations: WB, Western blot analysis, IHC, immunohistochemistry; IF, immunofluorescence.

### Quantitative real‐time PCR (qRT‐PCR)

The qRT‐PCR technique was previously described in another study 16. Reverse transcription was performed using the ImProm‐II Reverse Transcriptase Kit (Promega, Madison, WI, USA) according to the manufacturer's instructions. qRT‐PCR analysis was performed using GoTaq qPCR Master Mix (A6001, Promega). mRNA levels were normalized to those of GAPDH and GUSB. Changes in the transcript level were calculated using the 2^−ΔΔCT^ method 17. Complementary deoxyribonucleic acid (cDNA) was amplified using primers indicated in Table 4. cDNA was amplified using the Rotor‐Gene™ 6000 Real‐Time PCR Machine (Qiagen GmbH, Hilden, Germany).

**Table 4:** Primers used for qRT‐PCR

Primer	Forward	Reverse
*GUSB*	5′‐CAAGGGGTCAATAAGCACGA‐3′	5′‐TCTGAGTAGGGATAGTGGCT‐3′
*GAPDH*	5′‐CAAGGACACTGAGCAAGAGA‐3	5′‐GCCCCTCCTGTTATTATGGG‐3′
*CYP11A1*	5′‐GGGCACTTTGGAGTCAGTTT‐3′	5′‐CGGTCTTTCTTCCAGGCATC‐3′
*HSD3B1*	5′‐GCTGCTGCACAGGAATAAAG‐3′	5′‐GCCTGCTTCGTGACCATATT‐3′
*CYP17A1*	5′‐ACACCTAATGCCAAGTTCCC‐3′	5′‐AGGCGAAGAGAATAGATGGG‐3′
*StAR*	5′‐ACACCCCAAAGAAGGCATAG‐3′	5′‐GCTGAATCCCCCAAACTTCT‐3′
	QuantiTect Primer Assay (Qiagen)	
*DNM2*	NM_001039520, bp 3579	
*OCLN*	NM_008756, bp 3192	
*GJA1*	NM_010288, bp 3105	
*TJP1*	NM_001163574, bp 6891	
*F11R*	NM_172647, bp 2482	

### Histological examination

After the killing of animals, samples of testes were taken from each mouse for histological analysis as described previously 18, 19. Sections were stained with haematoxylin and eosin, mounted with coverslips and finally observed with a Leica DM5000 upright microscope (Leica Microsystems, Heidelberg, Germany). Two independent observers (F.C and F.R) examined the specimens on two separate occasions, in a blind manner, using coded slides without knowing their source.

For the histological evaluation, 10 sections which had a 20‐μm distance from each other were observed with the light microscopy and the images were taken at ×40 magnification.

### Immunofluorescence analysis

For immunofluorescence, deparaffinized sections of 4–5 μm were incubated in the antigen unmasking solution (10 mM tri‐sodium citrate, 0.05% Tween‐20) for 8 min. at 75°C and treated with a blocking solution (3% BSA in PBS) for 30 min. Next, the primary antibody (Table 3) was applied, and the slides were incubated in a humidified chamber overnight at 4°C. Then, the sections were incubated for 1 hr at 23°C with a conjugated secondary antibody (anti‐rabbit IgG–FITC antibody produced in goat, F0382, Sigma‐Aldrich; antimouse IgG–TRITC antibody produced in goat, T5393, Sigma‐Aldrich). Nuclei were stained with Hoechst stain solution (1:1000, Hoechst 33258; Sigma‐Aldrich). The slides were treated with PermaFluor Mountant (Thermo Fisher Scientific, Inc. Waltham, MA, USA) and cover‐slipped. The images were captured using a Leica Confocal Microscope TCS SP8 (Leica Microsystems).

### Immunohistochemistry analysis

For immunohistochemical analysis, serial sections (4–5 μm) were incubated in an antigen unmasking solution for 8 min. at 75°C. Then, the MACH1 kit (M1u539 g; Biocare, Concord, CA, USA) was used according to the manufacturer's instructions. The sections were incubated with the primary antibody in a humidified chamber overnight at 4°C. The following day, the sections were incubated for 1 hr with the secondary antibody. Finally, the slides were cover‐slipped, and images were captured with a Leica DM5000 upright microscope.

### Statistical analyses

A one‐way anova followed by a Bonferroni post hoc test for multiple comparisons was performed as an appropriate analysis for the data. All statistical analyses were performed using the GraphPad PrismTM 4.0 program (GraphPad Software Inc., San Diego, California, USA). All data are presented as the mean ± S.D., and the level of statistical significance was set at *P* < 0.05.

## Results

### Body weight

**Figure 1:**
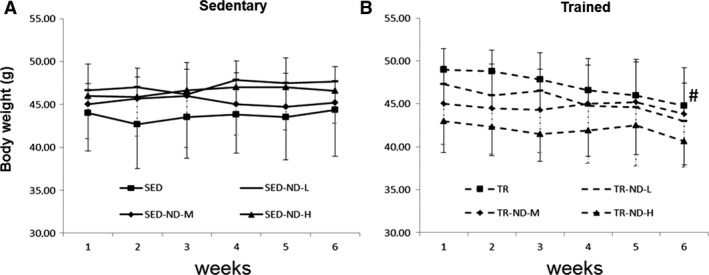
Functional effects of endurance exercise on body weight. Changes in body weight over time. All mice were weighed every week. Horizontal axis: time of training (weeks). Vertical axis: body weight (g). (**A**): normal control mice (SED), sedentary low dose of ND (SED‐ND‐L), sedentary medium dose of ND (SED‐ND‐M), sedentary high dose of ND (SED‐ND‐H). (**B**): trained control mice (TR), trained low dose of ND (TR‐ND‐L), trained medium dose of ND (TR‐ND‐M) and trained high dose of ND (TR‐ND‐H). Data are presented as the mean ± S.D. # significantly different from TR first week mice (*P* < 0.05).

All of the trained mice successfully completed the 6‐week training programme without the aid of electric shock incentive, and no injuries were sustained throughout the training. The mice were weighed at the beginning of the experiment and every week thereafter. Body weight of mice from all groups is shown in Figure 1. No difference in body weight was observed between the trained groups (TR, TR‐ND‐L, TR‐ND‐M and TR‐ND‐H) and the sedentary groups (SED, SED‐ND‐L, SED‐ND‐M and SED‐ND‐H) after 6 weeks of training. A statistical analysis was also carried out within the same group. Only the TR group showed a reduction in body weight after 6 weeks of training compared with the body weight at the beginning of the experimental protocol (*P* < 0.05) (Fig. 1B).

### Effect of ND administration on testosterone biosynthesis

**Figure 2:**
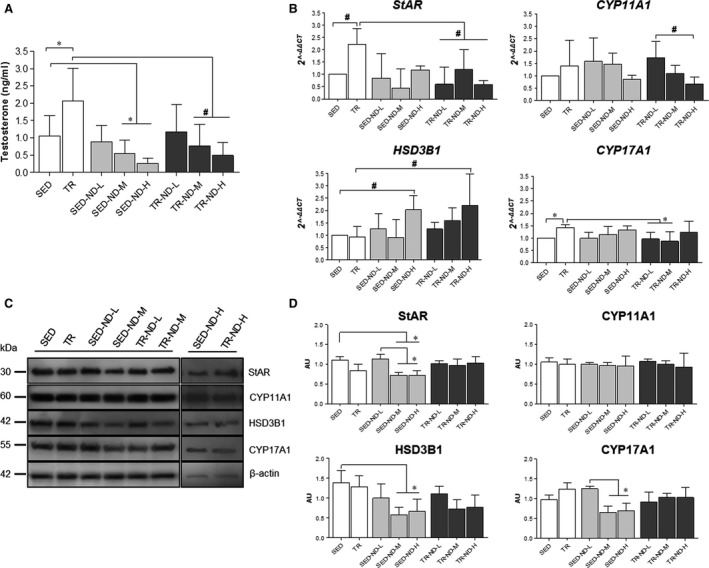
Effect of ND on testosterone secretion and steroidogenic gene/protein expression. (**A**): Measurement of testosterone level performed with liquid chromatography–mass spectrometry. Vertical axis: testosterone levels (ng/ml). Horizontal axis: mice groups. Normal control mice (SED), sedentary low dose of ND (SED‐ND‐L), sedentary medium dose of ND (SED‐ND‐M), sedentary high dose of ND (SED‐ND‐H), trained control mice (TR), trained low dose of ND (TR‐ND‐L), trained medium dose of ND (TR‐ND‐M) and trained high dose of ND (TR‐ND‐H). (**B**): qRT‐PCR evaluation of *StAR*,*CYP11A1*,*HSD3B1* and *CYP17A1* gene expression after ND administration and/or endurance training. The graphs show normalization with the reference genes, according to the Livak method (2^−∆∆CT^). Vertical axis: 2^−∆∆CT.^ Horizontal axis: mice groups. (**C**): representative cropped blots for StAR (30 kDa), CYP11A1 (60 kDa), HSD3B1 (42 kDa) and CYP17A1 (55 kDa). The gels were run under the same experimental conditions and β‐actin was used as the internal control. (**D**): relative expression levels of StAR, CYP11A1, HSD3B1 and CYP17A1. Vertical axis: arbitrary units (AU). Horizontal axis: mice groups. Data are presented as the mean ± S.D. *P < 0.05; #*P* < 0.01.

To determine whether ND stimulation affected testosterone production, measurement of testosterone level in serum was taken using liquid chromatography–mass spectrometry. The hormone levels were significantly higher in response to endurance training in the TR group compared with the SED group (*P* < 0.05). We observed a significant decrease in testosterone production in TR‐ND‐M and TR‐ND‐H groups compared with the TR group (*P* < 0.01). Moreover, testosterone levels in serum were significantly lower in SED‐ND‐M and SED‐ND‐H groups compared with the SED group (*P* < 0.05) (Fig. 2A).

To determine variation in gene expression, we performed qRT‐PCR for StAR, CYP17A1, HSD3B1 and CYP11A1 genes (Fig. 2B). The results showed that the expression of StAR and CYP17A1 mRNA increased significantly in the TR group compared with SED, TR‐ND‐L and TR‐ND‐M groups (*P* < 0.05). The expression of StAR mRNA increased significantly in the TR group compared with the TR‐ND‐H group (*P* < 0.01). Moreover, the expression of HSD3B1 mRNA increased significantly in SED‐ND‐H and TR‐ND‐H groups, respectively, compared with SED and TR groups (*P* < 0.01). Finally, the expression of CYP11A1 mRNA decreased significantly in the TR‐ND‐H group compared with the TR‐ND‐L group (*P* < 0.01).

Lysates of testes were analysed by Western blotting analysis in all experimental groups to verify the effects of ND administration and training on expression levels of proteins involved in testosterone synthesis (Fig. 2C). Our results showed that the levels of StAR decreased significantly in SED‐ND‐M and SED‐ND‐H groups compared with SED and SED‐ND‐L groups (*P* < 0.05) (Fig. 2D). The levels of CYP17A1 decreased significantly in SED‐ND‐M and SED‐ND‐H groups compared with the SED group (*P* < 0.05). Moreover, the levels of HSD3B1 decreased significantly in SED‐ND‐M and SED‐ND‐H groups compared with the SED group (*P* < 0.05). Finally, Western blotting analysis for CYP11A1 protein level did not show any significant differences between the groups.

### Histological analysis

**Figure 3:**
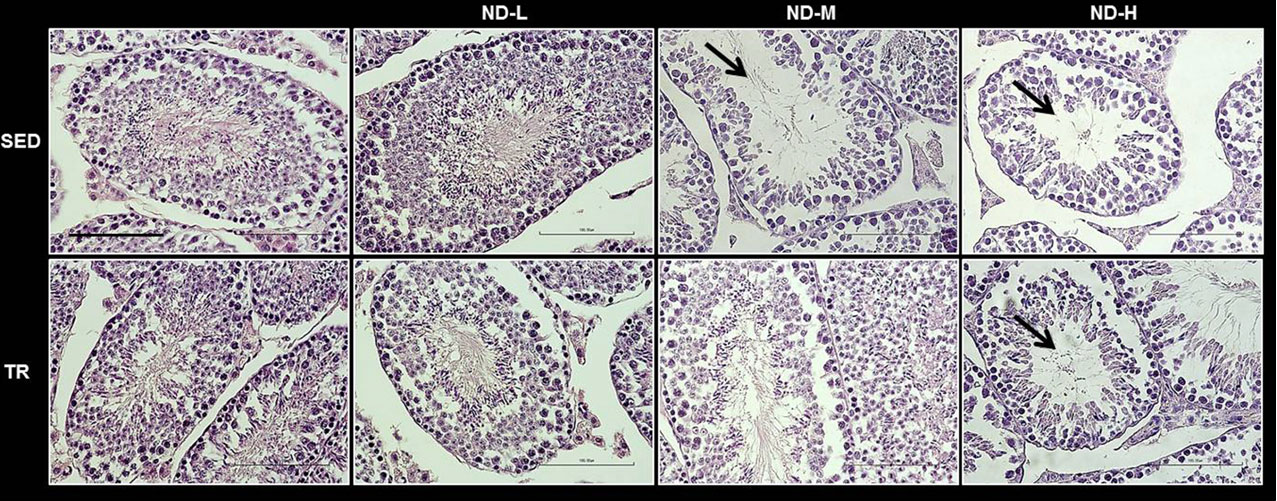
Representative photomicrographs of testis sections stained with haematoxylin–eosin. Testis histology of normal control mice (SED), trained control mice (TR), low dose of ND (ND‐L), medium dose of ND (ND‐M) and high dose of ND (ND‐H). The photomicrographs showed degenerative changes and disorganization of the normal histology of testes with an incomplete germ cell maturation of seminiferous tubules (see arrows). Bar = 100 μm for all panels.

The histological analysis carried out on mice testis samples showed alterations in the normal histological structure in SED‐ND‐M, SED‐ND‐H and TR‐ND‐H groups. In these groups, some seminiferous tubules showed degenerative changes and disorganization with an incomplete germ cell maturation (Fig. 3). Cells that resemble primary spermatogonial cells with irregular and dense nuclei were present in the lumen of some tubules while there was a loss of spermatids and mature germ cells. The samples also showed interstitial Leydig cell atrophy. Normal histological structure of seminiferous tubules with complete spermatogenic series and normal maturation was observed in other groups.

### Effect of ND stimulation on gene expression levels of BTB components

**Figure 4:**
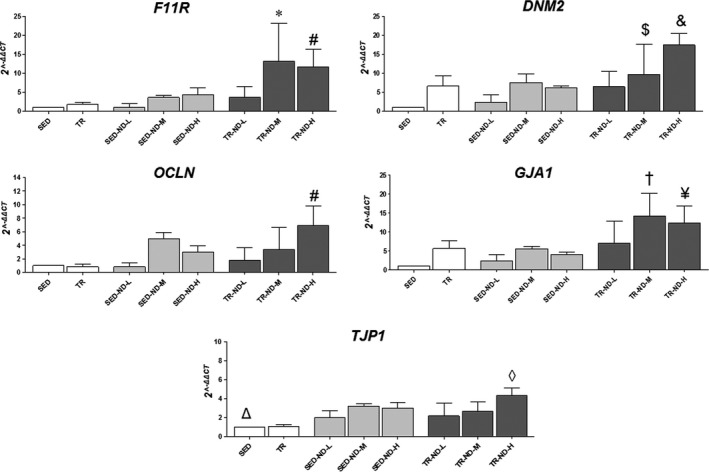
Effect of ND on gene expression levels of BTB components. qRT‐PCR evaluation of *Junctional adhesion molecule A* (*JAM1*), *Dynamin‐2* (*DNM2*), *Occludin* (*OCLN*), *Gap junction alpha‐1* (*GJA1*) and *TJP1* gene expression after ND administration and/or endurance training. The graphs show normalization with the reference genes, according to the Livak method (2^−∆∆CT^). Vertical axis: 2^−∆∆CT^. Horizontal axis: mice groups. Normal control mice (SED), sedentary low dose of ND (SEDND‐L), sedentary medium dose of ND (SED‐ND‐M), sedentary high dose of ND (SED‐ND‐H), trained control mice (TR), trained low dose of ND (TR‐ND‐L), trained medium dose of ND (TRND‐M) and trained high dose of ND (TR‐ND‐H). Data are presented as the mean ± S.D. *vs SED, TR, TR‐ND‐L and SED‐ND‐M (*P* < 0.05); #vs SED, TR, TR‐ND‐L and SED‐ND‐H (*P* < 0.01); $vs SED (*P* < 0.05); &vs SED, TR, TR‐ND‐L, TR‐ND‐M and SED‐ND‐H (*P* < 0.01); †vs SED and SED‐ND‐M (*P* < 0.05); ¥vs SED and SED‐ND‐H (*P* < 0.05); Δvs SED‐ND‐M, SED‐ND‐H, TR‐ND‐H (*P* < 0.05); ◊vs TR and TR‐ND‐L (*P* < 0.05).

To better evaluate the effect of ND stimulation on BTB dysregulation at the genetic level, qRT‐PCR analysis was performed for *Junctional adhesion molecule A (JAM1)* also called *F11 receptor (F11R)*,* Dynamin‐2 (DNM2)*,* Occludin (OCLN)*,* Gap junction alpha‐1* (*GJA1* or *connexin‐43*) and *Tight junction protein‐1* (*TJP1*or *ZO‐1*). The results obtained are shown in Figure 4. *F11R* gene levels were elevated in the TR‐ND‐M group compared with SED, TR, TR‐ND‐L and SED‐ND‐M groups (*P* < 0.05). Moreover, *F11R* and *OCLN* gene expression levels were significantly higher in the TR‐ND‐H group compared with SED, TR, TR‐ND‐L and SED‐ND‐H groups (*P* < 0.05). TR‐ND‐M and TR‐ND‐H groups showed significantly higher levels of *DNM2* compared, respectively, with SED and TR, TR‐ND‐L, TR‐ND‐M, SED and SED‐ND‐H groups (*P* < 0.05). Furthermore, qRT‐PCR analysis revealed that ND stimulation significantly enhanced *GJA1* levels in the TR‐ND‐M group compared with SED and SED‐ND‐M groups. In the TR‐ND‐H group, *GJA1* expression levels were higher compared with SED and SED‐ND‐H groups (*P* < 0.05). *TJP1* expression significantly increased in SED‐ND‐M, SED‐ND‐H and TR‐ND‐H groups compared with the SED group (*P* < 0.01). Finally, TR‐ND‐H group showed elevated levels of the same gene compared with TR and TR‐ND‐L groups (*P* < 0.01).

### Immunofluorescence analysis

**Figure 5:**
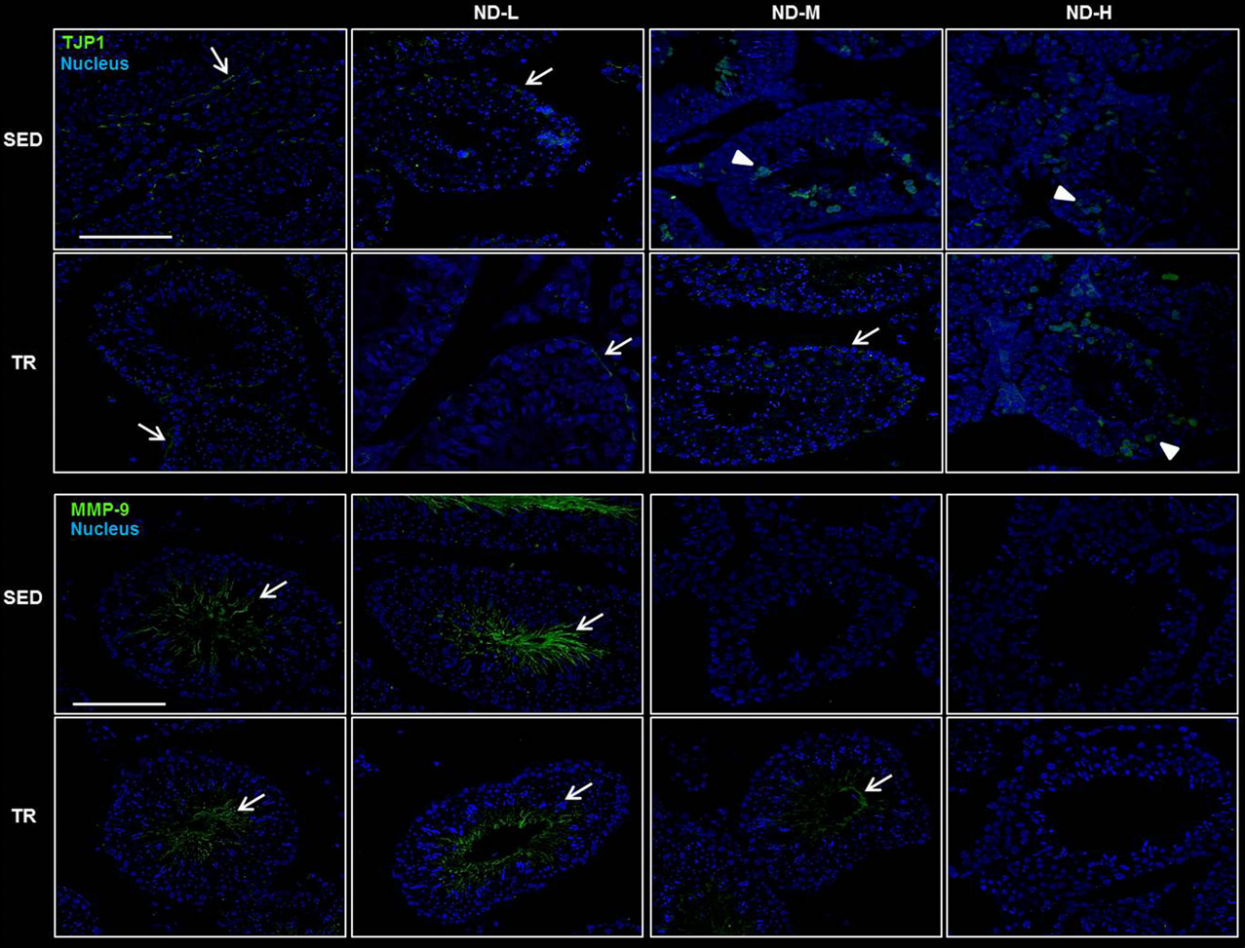
Representative images of immunofluorescence stain for TJP1 and MMP‐9 in testis sections. Testis histology of normal control mice (SED), trained control mice (TR), low dose of ND (ND‐L), medium dose of ND (ND‐M) and high dose of ND (ND‐H). The images above show TJP1 immunoreactivity distributed at the level of the BTB visibly as a line between adjacent Sertoli cells (see arrow) whereas arrow head indicates TJP1 signal in the cell cytoplasm at the basal and adluminal compartment of the seminiferous tubules. The images below show metalloproteinase‐9 (MMP‐9) immunoreactivity distributed pre‐eminently in the flagella of spermatocytes at the adluminal compartment of seminiferous tubules (see arrow). Bar = 100 μm for all panels.

**Figure 6:**
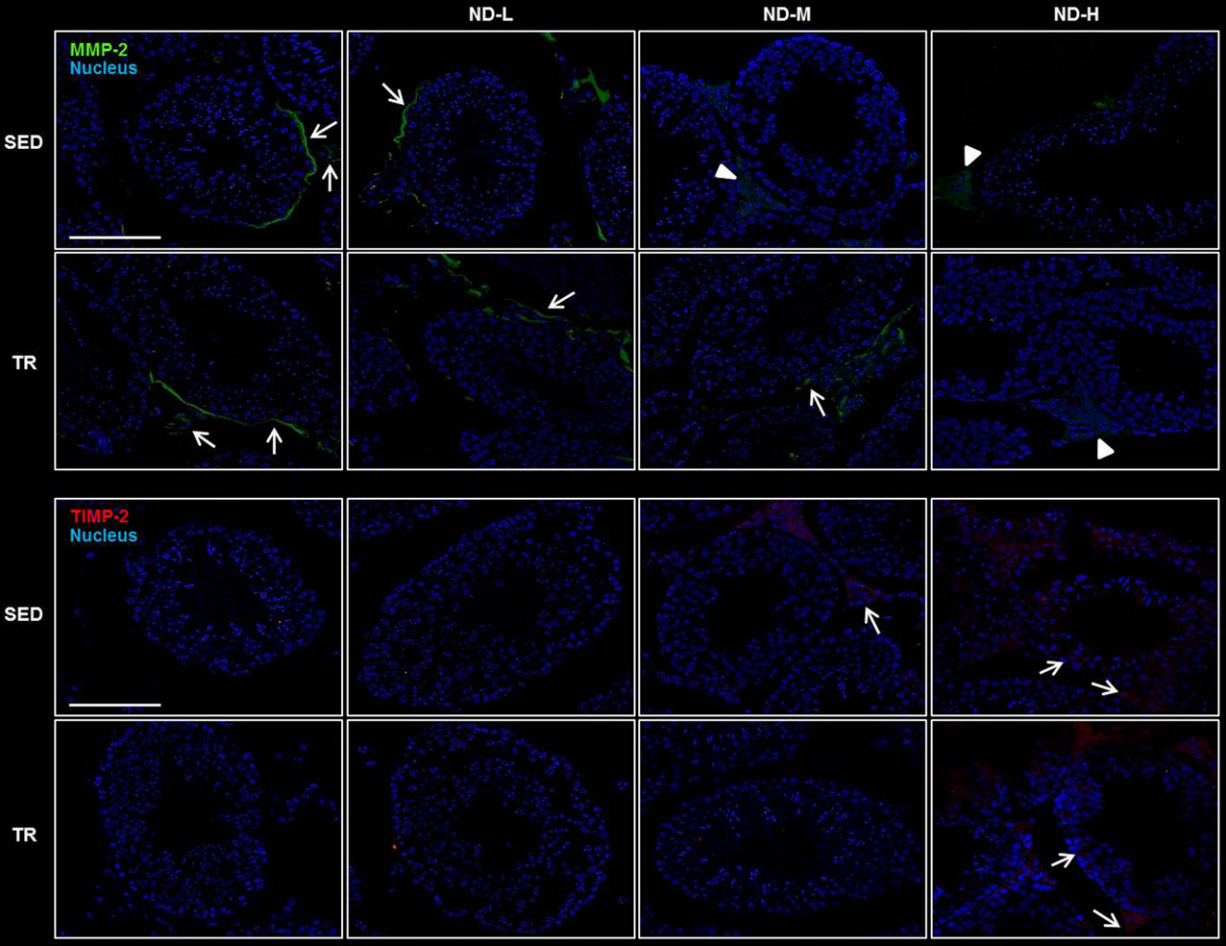
Representative images of immunofluorescence stain for MMP‐2 and TIMP‐2 in testis sections. Testis histology of normal control mice (SED), trained control mice (TR), low dose of ND (ND‐L), medium dose of ND (ND‐M) and high dose of ND (ND‐H). The images above show MMP‐2 immunoreactivity distributed at the level of the basal lamina of the seminiferous tubules as well as in the cytoplasm and cell membrane of Leydig cells (see arrow) whereas arrow head indicates MMP‐2 distribution in the cytoplasm of Leydig cells. The images below show the tissue inhibitor of metalloproteinase‐2 (TIMP‐2) immunoreactivity distributed at the level of the basal lamina of the seminiferous tubules and in the cytoplasm of Leydig cells (see arrow). Bar = 100 μm for all panels.

Blood–testis barrier integrity was examined using immunofluorescence staining for TJP1 as a marker of tight junction proteins (Fig. 5). In testes of SED, TR, SED‐ND‐L, TR‐ND‐L and TR‐ND‐M groups, TJP1 was distributed only at the base of seminiferous tubules while in SED‐ND‐M, SED‐ND‐H and TR‐ND‐H groups, testes showed altered distribution patterns of the protein. In fact, ND seems to induce TJP1 internalization and localization in the seminiferous tubule cytoplasmic compartment. Metalloproteinase‐9 (MMP‐9) immunoreactivity was detected pre‐eminently in the flagella of spermatocytes at the adluminal compartment of seminiferous tubules in SED, TR, SED‐ND‐L, TR‐ND‐L and TR‐ND‐M groups (Fig. 5). Indeed, SED‐ND‐M, SED‐ND‐H and TR‐ND‐H groups did not yield a signal in response to this protease. Immunofluorescence localization of MMP‐2 was detected in the basal lamina of the seminiferous tubules as well as in the cytoplasm and cell membrane of Leydig cells, with a granular distribution resembling probable secretion bodies. This tissue distribution was demonstrated in SED, TR, SED‐ND‐L, TR‐ND‐L and TR‐ND‐M groups while in the other groups MMP‐2 was present with a widespread granular distribution in the cytoplasm of Leydig cells (Fig. 6). Finally, the tissue inhibitor of metalloproteinase‐2 (TIMP‐2) immunopositivity was inversely correlated to MMP‐2 immunoreactivity (Fig. 6).

### Immunohistochemical analysis

**Figure 7:**
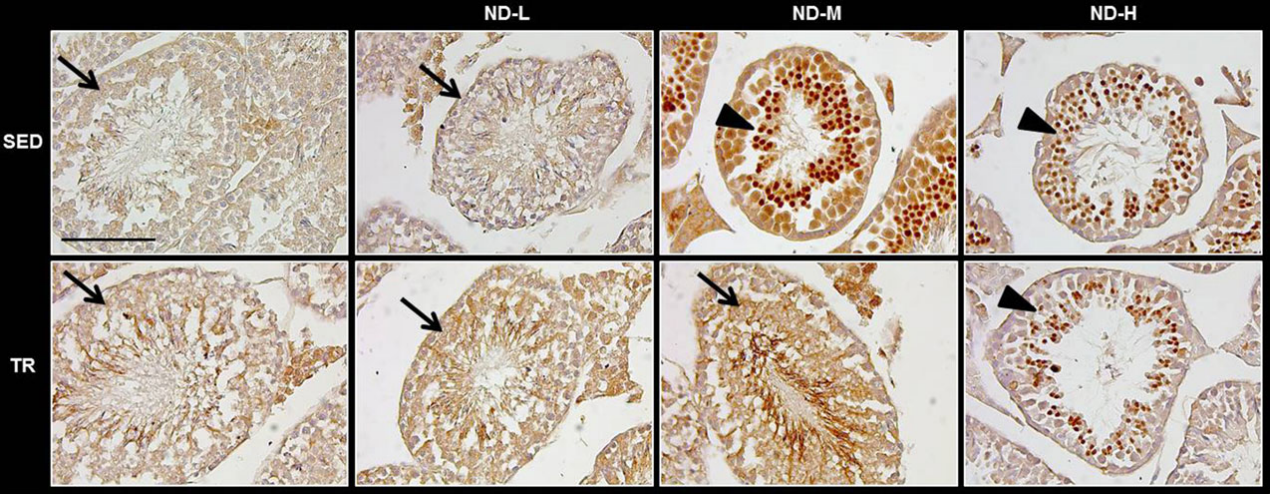
Representative images of immunohistochemical stain for MUC1 in testis sections. Testis histology of normal control mice (SED), trained control mice (TR), low dose of ND (ND‐L), medium dose of ND (ND‐M) and high dose of ND (ND‐H). Moderate signal of mucin 1 (MUC1) immunoreactivity inside the cytoplasm of some germ cells (arrow). Intense signal of MUC1 immunoreactivity in the nuclei of spermatids of seminiferous tubules (arrow head). Bar = 100 μm for all panels.

To determine the tissue distribution of mucin 1 (MUC1), immunohistochemistry was carried out (Fig. 7). The observation of the testis specimens under optical microscope revealed the presence of MUC1 in the nuclei of spermatids of many seminiferous tubules in SED‐ND‐M, SED‐ND‐H and TR‐ND‐H groups. The protein immunoreactivity was strong and intense. The other groups showed MUC1 immunoreactivity inside the cytoplasm of some germ cells, but the signal strength was moderate. Moreover, in all groups the immunohistochemical staining revealed the expression of MUC1 in the cytoplasm of Leydig cells.

## Discussion

In a previous study, we demonstrated that ND treatment of Leydig cells interferes with the biosynthesis of testosterone in a dose increase‐dependent fashion 13. As a consequence of the results obtained *in vitro*, here an animal model was utilized to better understand the side effects of ND administration in sedentary and trained mice.

The results obtained showed that endurance training increased serum testosterone levels in the TR group compared with the control group. The increased hormonal level following aerobic training may represent a physiological adaptation, confirming the effectiveness of our training protocol in accordance with another study 20, 21. Moreover, ND administration at middle to high doses decreased serum testosterone levels in sedentary and trained mice, allowing us to hypothesize that ND may, *via* an unknown mechanism, interfere with testosterone biosynthesis. The results obtained in this study are consistent with the findings of another study which demonstrated persistently low testosterone levels throughout the duration of ND treatment. This may possibly be attributed to negative feedback mechanisms, with reduced endogenous testosterone secretion 22.

It has been established that exercise and high doses of ND may influence the hypothalamic–pituitary–gonadal axis 23, 24. In particular, it has been suggested that chronic administration of ND decreases levels of LH and follicle‐stimulating hormone, which then lead to decreased endogenous testosterone production and decreased spermatogenesis in male rats 23, 24. Low levels of LH may contribute to low levels of testosterone production as LH stimulates testosterone production in Leydig cells, and this could explain the results obtained in this study. This therefore demonstrates that testosterone secretion decreases in mice administered with middle to high doses of ND. These changes were accompanied by modifications in expression levels of the main enzymes involved in testosterone production as ND acts as a competitive inhibitor of testosterone 25. Our results showed a significant decrease in StAR and CYP17A1 gene levels in mice trained and administered with the ND group compared with the TR group. Moreover, we observed a significant increase in HSD3B1 gene levels in mice administered with a high dose of ND, in both sedentary and trained mice compared with SED and TR groups, respectively. A significant increase in StAR and CYP17A1 gene expression levels in trained mice without ND administration, compared with the SED group, was also observed. These results are in agreement with previous findings 21 that demonstrated that endurance training and conjugated linoleic acid administration increased the gene expression of CYP17A1. On the contrary, ND administration decreased expression levels of this gene and thus testosterone production. StAR overexpression has already been associated with an increment in testosterone production 26 while to the best of our knowledge, a reduction in CYP17A1 levels and/or expression after ND stimulation has never been previously reported in the literature. Bjelic *et al*. 6 reported a dramatic decrease in CYP11A1 and CYP17A1 gene levels while the levels of HSD3B increased in Leydig cells after testosterone enanthate treatment. We hypothesize that high ND concentrations may interfere with CYP17A1 gene transcription through unknown mechanisms, which indirectly affect testosterone production. It is well known that the regulation of steroidogenic gene transcription is complex, involving a broad range of different transcription factors. For example, in Leydig cells, StAR, CYPs, HSD3B and cAMP regulate the promoter's activity, involving primarily CREB, NUR77, GATA4 and SF1. It is also modulated by other transcriptional cofactors as well as repressors 27, 28, 29. As stated above, we provided data supporting the hypothesis that ND might regulate testosterone production by inhibiting CYP17A1 gene/protein levels although further long‐term studies are warranted in order to elucidate the underlying molecular mechanism involved.

Considering the data obtained for testosterone production after ND administration and its essential role in male fertility, we investigated whether AAS induces morphological alterations in testes. The histological analysis of testes from sedentary and trained mice ND administered showed a disorganized histological structure of the seminiferous tubules with an incomplete germ cell maturation that might lead to impaired spermatogenesis. It is well known that the BTB integrity is crucial for spermatocyte differentiation, and we asked whether ND stimulation may induce modifications at the gene expression level of some components of the barrier. The results obtained demonstrated an increase in the gene levels coding TJ‐integral membrane proteins, such as *OCLN, F11R* and their common adaptor *TJP1* in trained mice administered with middle and high ND doses. Moreover, in the same groups, we observed elevated gene expression levels for *DNM2* encoding the homologous protein associated with ES. Among the D‐GJs of the BTB, we examined the gene expression levels of *GJA1* which showed a trend similar to the other molecules investigated. TJP1 tissue distribution was further investigated by immunofluorescence staining as this is a key protein that mediates Sertoli cell–cell adhesion. This function is facilitated by the fact that the protein is anchored in F‐actin bundles, conferring a degree of impermeability to the BTB 30. In control testes, TJP1 output fluorescent signal was visibly accentuated at the level of the BTB as a line between adjacent Sertoli cells. Following ND treatments, TJP1 signal was mainly distributed in the cell cytoplasm at the basal compartment, consistent with the location of the BTB. These positive cells resembled Sertoli cells. Moreover, TJP1 immunoreactivity was also observed in the cytoplasm of some cells at the adluminal compartment of the seminiferous tubules. Interestingly, in TR‐ND‐M group, TJP1 distribution is similar to control groups suggesting a protective effect of exercise. The altered distribution of TJP1 suggests that this protein can be targeted by ND as part of the drug's side‐effect profile, *that is* the decrease in testosterone levels. It has been demonstrated that testosterone promotes the integrity of the BTB *in vivo* 31, 32 and *in vitro* by enhancing the recycling of internalized proteins to the cell surface and relocating these proteins to reassemble and seal the barrier 33, 34. Moreover, TJP1 delocalization could reflect impaired functionality of the basal epithelium as it is associated with carcinoma *in situ* 35. Similar results were obtained in rabbits fed with cholesterol‐rich diet in which the disruption of the BTB and the appearance of unconventional TJP1 in endosomes were correlated with impaired spermatogenesis and infertility 36.

In our study, ND stimulation resulted in deregulated MMP‐9, MMP‐2 and TIMP‐2 expression. These proteases degrade components of the extracellular matrix and basement membranes in a zinc‐dependent manner 37. MMP‐9 was detected on the flagella of spermatocytes in SED, TR, SED‐ND‐L, TR‐ND‐L and TR‐ND‐M while the immunofluorescence signal was absent in the other groups. This protease is essential for assessing semen quality 37. MMP‐2 regulates the migration of spermatogonia and spermatocytes *via* the degradation of components (laminin, collagen IV) of the BTB at stage VII and early VIII. This process promotes the disassembling of basal ES and the dissolution of apical ES after secretion by Sertoli cells 38. Moreover, MMP‐2 was shown to disrupt Sertoli–germ cell adhesion 39. The immunofluorescence experiments demonstrated that ND seems to regulate MMP‐2 secretion *via* the induction of TIMP‐2, with consequences on spermatocytes maturation and possibly male fertility.

We also investigated whether ND administration might induce alterations at the cellular surface level through the detection of MUC1. This protein is a component of the mucosal glycocalyx and has protective functions. It is associated with the testicular germ cell line and impaired spermatogenesis 40. A limited number of studies have reported data on the role of mucins in the genital tract of males, and there was also a variable form of glycosylation associated with maturation arrest at the level of spermatids and spermatocytes 40. The results obtained revealed moderate immunoreactivity for intracytoplasmic MUC1 of both germ cells and Leydig cells of mice testes from the sedentary and trained groups. Interestingly, MUC1 expression was detected in the nuclei of spermatids of many seminiferous tubules in sedentary mice administered with moderate to high doses of ND, and in trained mice administered with high ND doses. These results indicate a translocation of the protein from the cytoplasm to the nuclei. Importation of MUC1 into the nucleus is associated with diverse functions including regulation of transcription and cell proliferation, acting as an oncoprotein 41, 42.

## Conclusion

A comprehensive analysis of the data obtained in this study suggested that middle to high ND doses, in both trained and sedentary mice, induced diminished testosterone secretion due to alterations in its biosynthetic pathway. On the other hand, the morphological findings in testes also showed an impairment of the BTB at the periphery of the seminiferous tubules which may have triggered the maturation arrest of spermatocytes. ND administration induced diminished endogenous testosterone production with consequent impaired internalization and redistribution of the proteins constituting the TJ. These include TJP1 and probably all the other molecules associated with it, although their gene expression was clearly enhanced. The testosterone decrease may induce the deregulation of other constituent of the BTB, such as basal and apical ES, *via* the inhibition of MMP‐2 expression. This may therefore affect spermatocyte maturation. Moreover, MMP‐9 depletion and MUC1 overexpression may be considered as part of a complicated pathway leading to infertility and to the progression of carcinogenesis. This enforces the idea that ND abuse may well be considered as both a potential inducer of male infertility and a potential risk factor to a low endogenous bioavailable testosterone.

## Conflicts of interest

The authors have no conflict of interest to declare.
